# DNA Methylation in Rice: Mechanisms, Regulatory Roles, and Beyond

**DOI:** 10.3390/ijms26178454

**Published:** 2025-08-30

**Authors:** Ting Li, Wen-Jing Li, Jian-Hong Xu

**Affiliations:** 1 Department of Agronomy, College of Agriculture & Biotechnology, Zhejiang University, Hangzhou 310058, China; lt01214231296@163.com (T.L.); wjlitt@163.com (W.-J.L.); 2 Hainan Institute, Zhejiang University, Hainan Seed Industry Laboratory, Yazhou Bay Science and Technology City, Sanya 572025, China

**Keywords:** DNA methylation, growth and development, stress response, genetic improvement, rice

## Abstract

As a crucial aspect of epigenetic research, DNA methylation is fundamental to genomic stability, gene transcription regulation, and chromatin remodeling. Rice is a staple food source for roughly half of the world’s population. Therefore, optimizing rice yield and stress tolerance is vital for global food security. With the continuous advancement of DNA methylation detection technologies, studies have shown that DNA methylation regulates various rice growth and development processes, including root differentiation and grain development, through the dynamic equilibrium of de novo methylation, maintenance methylation, and demethylation. Furthermore, DNA methylation is crucial in the plant’s response to environmental stressors like high or low temperature, drought and salinity. The patterns of DNA methylation modifications are also closely linked to rice domestication and heterosis formation. Therefore, a comprehensive investigation of the DNA methylation regulatory network holds significant theoretical value for rice genetic improvement and molecular breeding. This review offers a systematic analysis of the molecular mechanisms and detection technologies of DNA methylation, as well as its regulatory roles in rice growth and development, stress responses, and other biological processes, aiming to provide a theoretical foundation for rice genetic improvement research.

## 1. Introduction

Classical genetics primarily focuses on the study of DNA sequence variations, emphasizing their regulatory impact on phenotypic variation traits. However, it encounters challenges in genetic phenomena, such as significant phenotypic variations despite consistent genotypes and the transgenerational inheritance of environmentally induced traits. In response to these limitations, epigenetics has emerged as a critical area of investigation aimed at addressing these complexities [1]. Epigenetics primarily explores heritable regulatory mechanisms that modulate gene expression without altering the DNA sequence, encompassing key aspects such as DNA methylation, histone modification, chromatin conformation remodeling, and non-coding RNAs [2].

DNA methylation represents a fundamental area of investigation within plant epigenetics. Currently, the scientific community has comprehensively elucidated the dynamic regulatory network of DNA methylation in the model plant *Arabidopsis*, with subsequent research progressively extending to various crop species [3,4,5]. Mechanistically, DNA methylation influences these processes by modulating DNA methylation patterns within gene and transposon regions, thereby affecting chromatin accessibility and gene transcriptional activity, ultimately regulating plant growth processes and stress response [6,7,8,9,10]. The *mddcc* mutant of *Arabidopsis*, a line engineered to be completely devoid of DNA methylation, not only exhibited extreme developmental defects, including dwarfism and failure to flower, but also a high frequency of active transposition events [11]. Meanwhile, exposure to cold stress induces the demethylase ROS1 to remove DNA methylation of promoter regions from genes associated with the CBF pathway, thereby activating the expression of downstream antifreeze genes [12].

Rice, as a staple crop, is critical for global food security [13]. Faced with the dual challenges of population growth and climate change, the crop scientific community has conducted extensive research on key biological processes, including yield formation, quality improvement, and disease resistance, leading to significant advances in genetic breeding and molecular regulation [14]. Relying on the established theoretical framework of DNA methylation in *Arabidopsis* and the ongoing refinement of the molecular regulatory network in rice, the scientific community has elucidated the critical roles of rice DNA methylation in growth, agronomic trait development, and stress response, thereby establishing a robust theoretical basis for epigenetic breeding in rice [15,16,17]. Concurrently, advancements in DNA methylation detection technologies and epigenomic techniques have provided crucial technical support for epigenetic breeding in rice. Specifically, in the field of epigenetic editing engineering, researchers have recently addressed challenges such as plant cell wall structures and low transformation efficiency, significantly improving editing efficiency and specificity to bolster the technical foundation for epigenetic breeding in rice [18,19]. Driven by both the elucidation of theoretical mechanisms and technological innovation, epigenetic breeding in rice has progressed from fundamental research to applied exploration, resulting in significant breakthroughs. The recent systematic investigation of rice cold stress by Song et al. represents a landmark achievement, proposing a novel “stress acclimation–epigenetic editing–stable trait inheritance” paradigm for epigenetic breeding [20]. Therefore, this review provides a comprehensive research progress of rice DNA methylation, aiming to inform strategies for genetic improvement of yield and quality, molecular breeding of stress-resistant varieties, and germplasm innovation in modern agriculture.

## 2. Molecular Mechanism of DNA Methylation

DNA methylation represents an epigenetic modification, catalyzed by methyltransferases, where SAM (S-adenosylmethionine) donates methyl groups to cytosine (C), generating 5mC (5-methylcytosine) [21]. The distribution of 5mC is typically enriched in transposable elements (TEs) and repetitive sequences within the genome. In mammals, DNA methylation is predominantly observed at symmetrical CG sites, while non-CG methylation (CHG and CHH, where H represents A, T, or C) is specifically distributed in certain cell types, including pluripotent stem cells, the brain, and oocytes [22,23,24,25]. In contrast, plants exhibit DNA methylation at all contents (CG, CHG, and CHH) [26]. Significant interspecies variations exist in DNA methylation and distribution patterns. In higher animal cells, 60-90% of CG sites are methylated [27], while the C methylation proportion in higher plant genomes can reach up to 43% [28]. Notably, DNA methylation in rice genome displays a fragmented distribution pattern, with an overall methylation level four times higher than that of *Arabidopsis* [29]. Plant DNA methylation is a dynamic regulatory process, with its modification status undergoing alterations in response to environmental changes and developmental stages [30]. The dynamic equilibrium of DNA methylation in plants is primarily regulated by three processes: de novo methylation, maintenance methylation, and demethylation [3].

### 2.1. De Novo Methylation

De novo methylation is defined as the process of establishing novel methylation patterns through the addition of methyl groups at specific genomic sites by DNA methyltransferases, facilitated by RdDM (RNA-directed DNA methylation) pathway in regions lacking prior methylation modifications [31].

**Figure 1:**
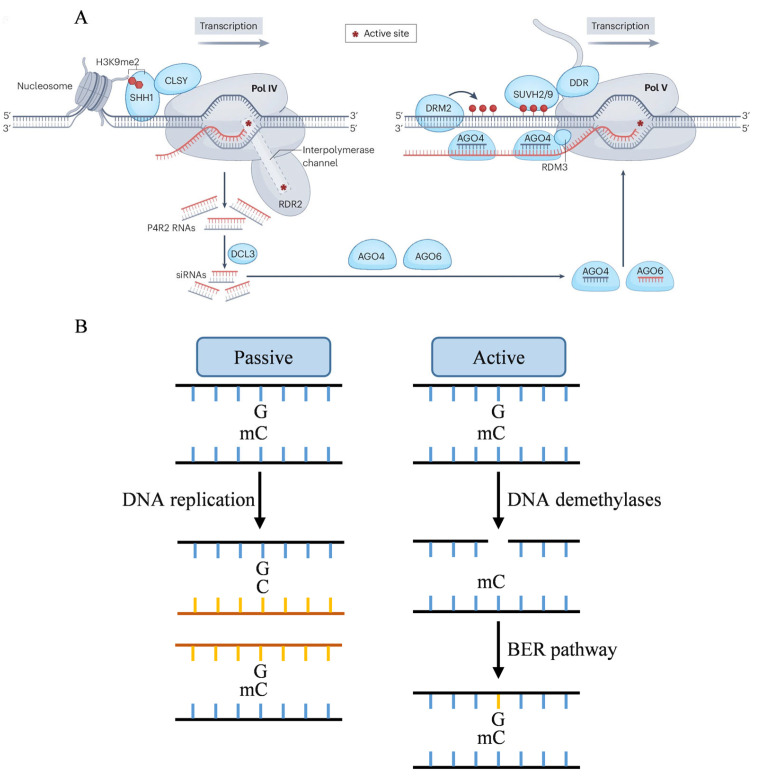
Mechanism of DNA de novo methylation and demethylation. (**A**) SHH1 exhibits preferential binds to demethylated H3K9me2, thereby facilitating the recruitment of DNA-dependent RNA Pol IV to a subset of RdDM loci through direct interaction with CLSY1 or CLSY2 chromatin remodeling factors. Pol IV and RDR2 form a holoenzyme that produces double-stranded precursor RNAs (P4R2 RNAs). These P4R2 RNAs undergo cleavage by DCL3 to produce 24-nt siRNA. One strand of the siRNA is subsequently bound and stabilized by AGO4 and AGO6 proteins. The siRNA-AGO complex is then recruited by Pol V transcripts through base pairing. SUVH2 and SUVH9, acting as methylcytosine binding proteins, bind to pre-existing methylcytosines, along with the DRD1-DMS3-RDM1 (DDR) complex. The siRNA-AGO complex, potentially in conjunction with other proteins, then recruits DRM2 to affect DNA methylation [52]. Reproduced with permission from Heng Zhang and Jian-Kang Zhu, Nature; published by Springer Nature, 2025. (**B**) DNA demethylation can occur passively during DNA replication. Active DNA demethylation is mediated by plant-specific DNA demethylases. The single-nucleotide gap will be filled with a unmethylated cytosine through BER pathway.

Studies have demonstrated that the RdDM de novo methylation pathway can be categorized into canonical (Figure 1A) and non-canonical types. The canonical pathway involves the histone-binding protein SHH1 (Suppressor of hairy-wing 1), which specifically recognizes histone H3K9me2, and the ZMP (Zinc finger protein), which specifically recognizes H3K4me0. These proteins cooperatively regulate the plant-specific RNA Pol IV (Polymerase IV) across various chromatin environments [32,33,34]. Following binding to these histone modification recognition proteins, specific POL IV subunits, such as the largest subunit NRPD1 (RNA polymerase IV subunit 1), transcribe ssRNA (single-stranded RNA) using specific chromatin loci as templates [35,36,37]. Simultaneously, other Pol IV subunits interact with RDR2 (RNA dependent RNA polymerase 2), employing ssRNA as a template to synthesize complementary strands, thereby forming dsRNA (double stranded RNA) [38,39,40,41]. Subsequently, dsRNA is cleaved by DCL3 (Dicer-like 3) into 24-nucleotide siRNA (small interfering RNA) [42,43]. The non-canonical pathway initiates with the cleavage of POL II transcripts mediated by miRNA (microRNA). After stabilization by SGS3 (Suppressor of gene silencing 3), the cleavage products recruit RDR6 to synthesize dsRNA, which DCL4 then processes to generate 21-nt siRNA [44,45,46,47]. siRNAs generated by both pathways undergo 3’ terminal methylation by the small RNA methyltransferase HEN1 (Hua enhancer 1) to maintain structural stability [48]. The modified siRNAs bind to AGO4 (Argonaute 4) or AGO6 to form the RISC (RNA-induced silencing complex), which targets DNA sequences based on complementary base pairing [49]. Furthermore, Pol V, after being recruited by DNA methylation, transcribes a 200-nt scaffold RNA. This scaffold RNA interacts with the RISC, and the cytosine residues of the target sequence are methylated by DRM2 (Domains rearranged methyltransferase 2) [50,51].

In *Arabidopsis*, DRM1/2 function via the RdDM pathway, primarily establishing methylation in CG and CHH contexts [53,54]. Meanwhile, CMT3 (Chromomethylase 3) localizes methylation sites via histone modification marks, predominantly responsible for methylation establishment in CHG context [55,56]. In rice, *OsDRM2*, the homologous of *Arabidopsis DRM2*, participates in the de novo methylation of CG and non-CG sites (Table 1) [57]. Rice has two copies of *CMT3* with distinct functions (Table 1). OsCMT3a is responsible for CHG methylation of TEs and centromeric region repetitive sequences, regulating rice development by suppressing TE transposition [58]. It also functions as the primary CHG methyltransferase in spermatocytes [59]. OsCMT3b, a rice-specific non-CG DNA methyltransferase, maintains non-CG methylation in specific GC-rich regions and regulates CHG methylation in microspores [59].

### 2.2. Maintenance Methylation

Methylation maintenance is the process of maintaining a stable methylation status during semi-conservative DNA replication, guided by the methylation pattern of parental strand. This is achieved through DNA methyltransferase, which add methylation modifications to the corresponding cytosine sites of the daughter strand [67].

In *Arabidopsis*, various DNA methyltransferases are responsible for maintaining the DNA methylation status of different cytosine sequences. CG methylation maintenance primarily relies on MET1 (Methyltransferase 1) [55,56,68]; CHG methylation maintenance is mainly dependent on CMT3; CHH methylation maintenance is primarily reliant on CMT2 and DRM1/2. Specifically, CMT2 maintains CHH methylation in heterochromatin, while DRM1/2 maintains CHH methylation in euchromatin or at the boundaries of long TEs [31,69,70]. DRM1/2 also maintains DNA methylation through the RdDM pathway. CMT3 forms a positive feedback regulatory loop with the histone methyltransferase SUVH4 (Suppressor of variegation 3-9 homolog 4), reinforcing CHG methylation maintenance. The catalytic activity of SUVH4 depends on the CHG methylation established by CMT3, while CMT3 recognizes the H3K9me2 modification catalyzed by SUVH4 through its BAH (Bromo adjacent homology) and chromo domains, thereby forming a positive feedback regulatory loop between DNA methylation and histone modification [71]. Similarly, CMT2 also maintains CHH methylation by recognizing H3K9me2-modified nucleosomes and collaborating with histone methyltransferases [72]. In the CG methylation maintenance pathway, the VIM1 (Variant in methylation 1) binds to MET1 via its SRA (Set and ring-finger-associated) domain, which enhances MET1 protein stability and precisely anchors MET1 to heterochromatin regions by binding to hypomethylated DNA and H3K9me2 modifications, thus establishing CG methylation [73].

In the rice genome, two homologous genes of *MET1* (*OsMET1a* and *OsMET1b*) have been cloned, exhibiting functional differentiation (Table 1) [60]. Both *OsMET1a* and *OsMET1b* are expressed in dividing cells-containing tissues, with *OsMET1b* showing significantly higher expression levels than *OsMET1a* [60]. In addition to *OsCMT3*, rice also has two unique *OsDDM1* (*OsDDM1a* and *OsDDM1b*) genes, which play a crucial role in CHG methylation and partial CG methylation of heterochromatic regions, and also participate in CHH methylation of gene regions, performing some functions of *OsCMT2* and *OsCMT3* (Table 1) [67,74].

### 2.3. DNA Demethylation

DNA demethylation can activate specific genes or reset the epigenetic status of the genome during development, encompassing passive and active demethylation (Figure 1B). Passive DNA demethylation arises when newly formed DNA strands fail to maintain their original methylation status post-replication, resulting in an unmethylated state. This process is often driven by factors such as the inhibition or loss of methyltransferase activity, methylation donor deficiency, and alterations in chromatin structure [75,76]. Active demethylation, on the other hand, is mediated by DNA demethylases, which excise methylated cytosine, followed by the synthesis of unmethylated cytosine through the BER (Base excision repair) pathway, ultimately resulting in DNA demethylation [77,78]. 

The active DNA demethylation process in plants can be delineated into three stages: substrate recognition, glycosidic bond hydrolysis, and DNA repair. Initially, DNA demethylases recognize 5mC sites and catalyze glycosidic bond hydrolysis, cleaving the bond between the base and deoxyribose, thereby removing 5mC and producing a base-free site. Subsequently, the BER pathway is activated, with the AP (Apurinic/apyrimidinic) endonuclease identifying the base site and cleaving the adjacent DNA strand. DNA polymerase then fills the gap with unmethylated cytosine, and finally, DNA ligase ligates the repaired DNA strand [79].

Unlike in plants, the active DNA demethylation process in animals is facilitated by the TET (Ten-eleven translocation) family of oxidases [80]. These enzymes catalyze the sequential oxidation of 5mC to 5hmC (5-hydroxymethylcytosine) [81], followed by 5fC (5-formylcytosine), and ultimately 5caC (5-carboxylcytosine) [82]. Subsequently, these oxidized derivatives are excised and replaced with unmethylated cytosine via the BER pathway [83,84]. Although plant genomes lack dioxygenases homologous to the mammalian TET family, the detection of 5mC oxidation products in species such as *Arabidopsis* [85], rice [86], and rye [87] suggests the presence of an oxidative demethylation mechanism in plants. Moreover, the heterologous expression of the catalytic domain of human TET3 in *Arabidopsis* induces the accumulation of 5hmC and 5fC, resulting in alterations in DNA methylation patterns [88]. Notably, these methylation modifications are stably inherited even after the transgene removal, demonstrating that exogenous TET enzymes retain oxidative functionality within plant systems.

Currently, researchers have identified eight DNA demethylase genes in rice, including four *OsROS1* (*Repressor of silencing 1*) genes (*OsROS1a*, *OsROS1b*, *OsROS1c*, and *OsROS1d*), two *Demeter-Like 3* (*OsDML3*) genes (*OsDML3a* and *OsDML3b*), and one each of *OsDML4* and *OsDML5* (Table 1) [89]. Studies have indicated that OsROS1a is involved in the demethylation process of CG and CHG in rice endosperm [62], while OsROS1a/b/c exert DNA demethylation effects in various genomic regions of gametes and zygotes [63]. Additionally, OsDML4 plays a crucial role in cytosine demethylation. In the *osdml4* mutant, the global methylation levels of CG, CHG, and CHH in seeds are significantly increased, impacting endosperm formation [65].

## 3. DNA Methylation Detection Methods

Epigenetics researchers have developed several DNA methylation sequencing methods to identify DNA methylation sites. The advent of BS (Bisulfite sequencing) represented the initial technology for DNA methylation sequencing [90]. Advances in sequencing technologies, particularly NGS (Next-generation sequencing), have enabled the development of various approaches like RRBS (Reduced representation bisulfite sequencing) [91], MeDIP-seq (Methylated DNA immunoprecipitation sequencing) [92], and WGBS (Whole-genome bisulfite sequencing) [93]. These technologies have found increasing application in plant science, from fundamental epigenetic mechanism studies to applications in crop breeding, stress response, and developmental regulation [94,95,96]. DNA methylation detection technologies are classified into two main categories based on their detection targets: targeted methylation detection technologies, which focus on specific genomic regions, and genome-wide methylation detection technologies, which analyze the entire genome.

**Figure 2:**
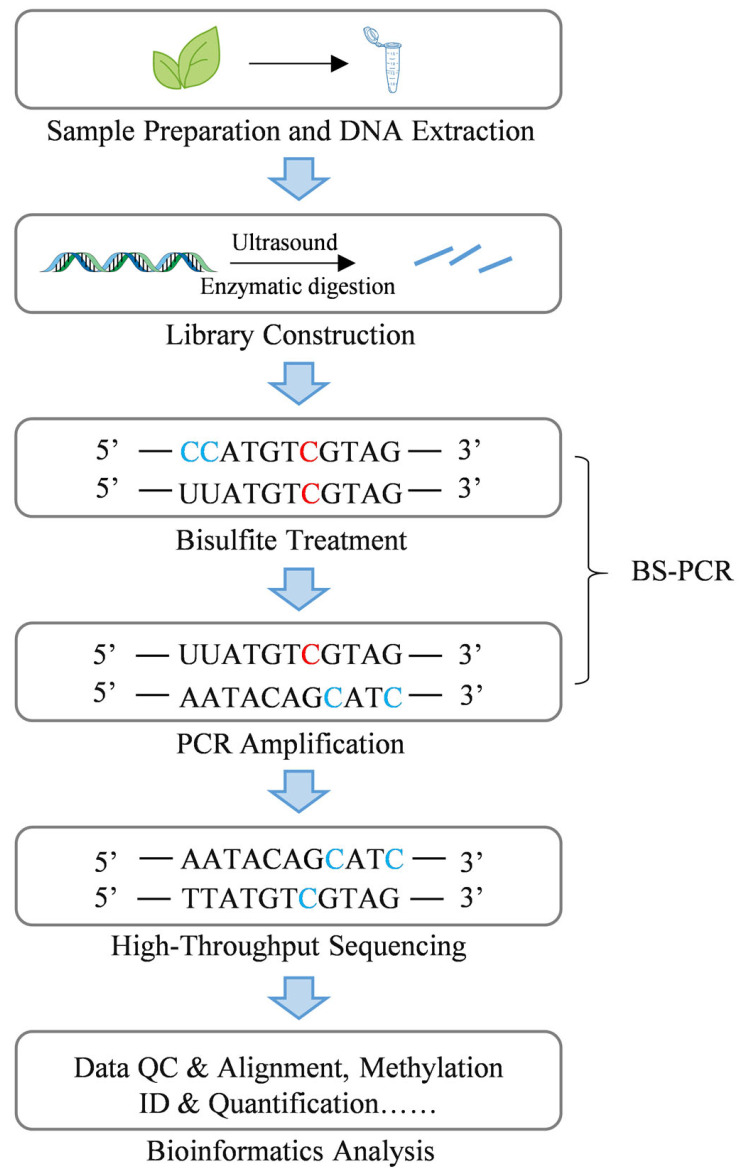
Principles and procedures of BS-PCR and WGBS. The select of appropriate samples and the extraction of high-quality DNA. The extracted DNA is then fragmented into smaller segments via ultrasound or enzymatic digestion, followed by adapter ligation for library construction. The library undergoes bisulfite treatment, which converts unmethylated cytosine (C) to uracil (U), while methylated cytosine (5mC) remains unchanged. PCR amplification is performed using the treated DNA as a template, where unmethylated C is replaced by thymine (T), and 5mC remains as C. the amplified products are then sequenced via high-throughput sequencing. Finally, bioinformatics analysis conducted, including data quality control, sequence alignment, and methylation qualitative and quantitative analysis. BS-PCR utilizes the bisulfite treatment and PCR amplification steps of WGBS to determines the DNA methylation status of specific loci through sequencing the amplified products. Unmethylated cytosine is denoted by the blue “C”, while methylated cytosine is denoted by the red “C”.

Targeted methylation detection technologies encompass a range of techniques, including MSP (Methylation-specific PCR), BSP (Bisulfite sequencing PCR), methylation pyrosequencing, TBS (Targeted bisulfite sequencing), MSRE-PCR (Methylation-sensitive restriction enzyme-PCR), MALDI-TOF (Matrix-assisted laser desorption/ionization time-of-flight mass spectrometry), oxBS-seq (oxidative Bisulfite sequencing), and TAB-seq (TET-assisted bisulfite sequencing) [97]. Among these, BS-PCR represents an early approach, utilizing bisulfite treatment to distinguish between unmethylated cytosine, which is converted to uracil (C→U), and 5mC, which remains unchanged. Following PCR amplification, U is read as thymine (T), whereas C that persist correspond to 5mC (Figure 2) [90]. Bisulfite treatment is a critical step in many other methylation detection methods. Pyrosequencing, after bisulfite treatment, enables real-time sequencing by detecing pyrophosphate release, directly quantifying the C/T ratio, which corresponds to 5mC/C [98]. TBS combines BS-PCR principles with high-throughput sequencing, allowing for the simultaneous analysis of multiple targeted loci [99]. Additionally, MALDI-TOF mass spectrometry, following bisulfite treatment and enzymatic digestion, quantifies methylation levels by detecting mass differences in DNA fragments [100]. To specifically detect 5hmC in plant genomes, oxBS-seq and TAB-seq were developed. oxBS-seq involves oxidizing 5hmC to 5fC, which is subsequently converted to U during bisulfite treatment, leaving only 5mC as C [91]. TAB-seq employs βGT (β-glucosyltransferase) to protect 5hmC by converting it to β-glucosylhydroxymethylcytosine (5gmC), while the TET enzyme oxidizes 5mC to 5caC, which is subsequently converted to U during bisulfite treatment, thus preserving 5gmC [101]. Additional targeted detection techniques including MSP, which bypass bisulfite treatment and determines methylation status through PCR amplification using primers specific to methylated or unmethylated sequences [102], and MSRE-PCR, which utilizes restriction enzymes that selectively cleave unmethylated sites, enabling the detection of 5mC via PCR amplification of uncleaved fragments containing methylated cytosines [103].

Genome-wide methylation detection technologies include WGBS, RRBS, XRBS (Extended representation bisulfite sequencing), methylation microarrays, MeDIP-seq, and hMeDIP-seq (Hydroxymethylated DNA immunoprecipitation sequencing) [97]. These methodologies enable the creation of plant epigenetic maps and facilitate large-scale screening. RRBS, originally developed with Sanger sequencing, employs restriction enzymes to digest genomic DNA, selectively enriches CpG-dense regions, followed by bisulfite conversion and sequencing for cost-effective genome-wide methylation analysis [91]. XRBS improves library preparation and sequencing protocols compared to RRBS, expanding CpG site coverage and enabling methylation status assessment across more gene regulatory elements [104]. WGBS involves high-throughput sequencing of the entire genomic DNA post-bisulfite treatment, providing comprehensive coverage of almost all C sites (Figure 2) [105]. It is considered the "gold standard" for methylation detection due to its genome-wide scope and single-base resolution. However, it has limitations, including high cost, data complexity, and the need for significant input DNA [106]. Besides bisulfite-dependent technologies, other genome-wide methylation detection techniques include methylation microarrays, MeDIP-seq and hMeDIP-seq. Methylation microarrays detect predetermined CpG sites (covering promoters, gene bodies, etc.) via probe hybridization, quantifying methylation levels based on fluorescent signal intensities. These arrays offer advantages such as high throughput, affordability, and straightforward data analysis, but are limited by probe dependency and potential hybridization biases [107]. MeDIP-seq uses 5mC-specific antibodies to immunoprecipitate DNA fragments containing 5mC, followed by high-throughput sequencing to map 5mC distribution [108]. hMeDIP-seq, based on MeDIP-seq, uses 5hmC-specific antibodies for genome-wide specific sequencing analysis of 5hmC, addressing the need to differentiate between 5hmC and 5mC modifications [109].

Various DNA methylation detection technologies previously documented have been implemented in rice research. The selection of detection methodologies in rice DNA methylation studies is contingent upon aligning research objectives (genome-wide analysis, specific gene validation, large-scale population screening) with technical attributes (coverage, resolution, cost). Among genome-wide methylation detection approaches, WGBS is frequently utilized [110,111]. WGBS serves as a fundamental tool for elucidating genome-wide methylation patterns, such as tissue-specific methylation variations and epigenetic modifications associated with domestication in rice [59,112]. Despite its associated costs, WGBS remains essential for in-depth mechanistic investigations [106]. Furthermore, rice, as a model species within the *Poaceae* family, characterized by a comapct and well-assembled genome, provides a crucial framework for methodologies that depend on high-quality genome alignment for WGBS [113,114]. In research focused on specific genes or genomic regions, the commonly employed targeted methylation detection technologies in rice are BSP and MSRE-PCR. BSP is widely used for ensure DNA methylation results due to its simplicity, low cost, and ability to accurately verify methylation dynamics of target genes [110,115,116]. MSRE-PCR, which obviates the need for bisulfite treatment, enables rapid qualitative preliminary screening of methylation differences in target genes, thereby rendering it advantageous for initial sample screening [117].

In rice research, genome-wide and targeted DNA methylation detection methodologies are commonly used complementarily [20,118]. The strategic integration of these techniques has significantly enhanced the understanding of epigenetic regulatory mechanisms involved in rice development, domestication, and stress responses, thereby providing essential epigenetic theoretical foundations for molecular breeding in rice.

## 4. Role of DNA Methylation in Rice Growth and Development

The rice life cycle, encompassing seed germination to the maturity of new seeds, is bifurcated into vegetative and reproductive phases. The vegetative growth stage initiates with seed germination, progressing through the seedling and tillering stage, culminating in the jointing stage. The reproductive growth stage commences with the booting stage, advancing through the heading and flowering stages, and concluding with seed maturity, which includes the milk, dough, and full maturity stages [119]. DNA methylation is a critical regulator throughout rice entire growth and development stages. The *osdrm2* mutant exhibits reduced genome-wide DNA methylation, it results in phenotypes, such as dwarfism, reduced tillering, and aberrant leave morphology in vegetative stage. While in the reproductive stage, it shows delayed heading, abnormal panicles and spikelets morphology, and complete sterility [57].

### 4.1. Vegetative Growth Stage

**Figure 3:**
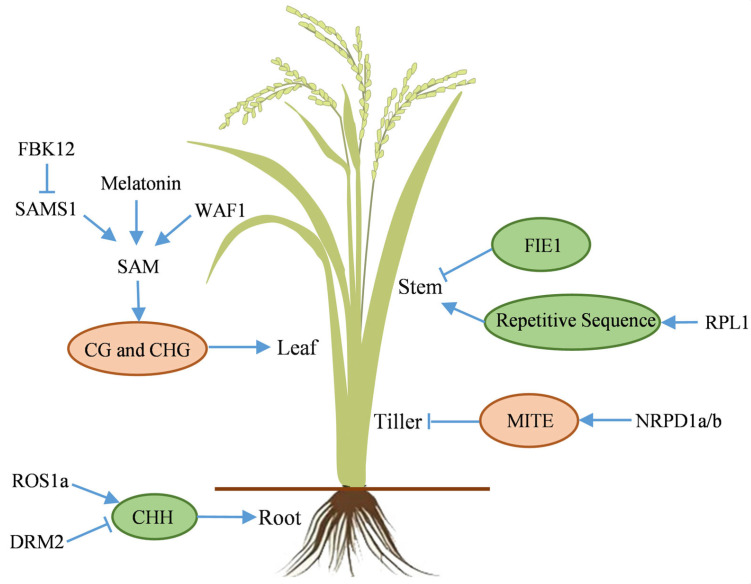
Mechanism of DNA methylation in the vegetative growth stage of rice. Blue arrows and T-bars indicate promotion and inhibition, respectively. Pink and green ovals represent increased and decreased DNA methylation, respectively. DRM2: Domains rearranged methyltransferase 2, FBK12: F-box protein containing a Kelch repeat motif, FIE1: Fertilization-independent endosperm 1, MITE: Miniature inverted-repeat transposable element, NRPD1a/b: Nuclear RNA polymerase D1a/b, ROS1a: Repressor of silencing 1, RPL1: Ribosomal protein l1, SAMS1: S-adenosylmethionine synthase 1, SAM: S-adenosylmethionine, WAF1: Wavy leaf1.

Rice vegetative organs, including root, stem, leaf, and tiller, function synergistically to support vegetative growth. The root system, consisting of primary, postembryonic crown, and lateral roots, facilitates water and mineral uptake. Primary roots are ephemeral, while the fibrous root system, composed of postembryonic crown and lateral roots, forms the primary absorptive network. Research indicates that CHH methylation influences the development of these root types, affecting transcriptome regulation. The activation of *OsROS1a* and the inhibition of *OsDRM2*, resulting in reduced DNA methylation, are essential for the expression of key functional genes (Figure 3) [120]. 

The stem provides structural support and serves as a conduit for nutrient transport. Stem development, consequently plant height, is regulated by DNA methylation via epigenetic mechanisms (Figure 3). The epigenetic allele *epi-DF* (*Epigenetic-dwarf*) of the *FIE1* (*Fertilization-independent endosperm 1*) gene, despite lacking DNA sequence alterations, exhibits hypomethylation in the promoter and 5’ region, accompanied with histone modification, leading to ectopic gene expression in stem internodes, ultimately causing plant dwarfing [116]. Additionally, mutation of the *rpl1* (*ribosomal protein l1*) gene increases methylation of repetitive sequences, disrupting hormone signaling and causing dwarf phenotype [121].

Leaf, the primary site of photosynthesis, converts light energy into chemical energy, supplying carbon sources and energy for plant growth. Rice leaf development is closely related to SAM levels. Melatonin deficiency induces premature leaf senescence, characterized by accelerated chlorophyll degradation, increased ROS (Reactive oxygen species) accumulation, and aberrant activation of senescence-related genes. Melatonin maintains DNA methyltransferase activity by stabilizing SAM levels (Figure 3). Melatonin deficiency impacts SAM synthesis, leading to a shortage of methyl donors and significant hypomethylation, particularly in TEs and promoters of genes involved in carbon metabolism and redox, ultimately resulting in elevated gene expression [122]. The F-box protein OsFBK12 (F-box protein containing a Kelch repeat motif 12) reduces SAM content by targeting and degrading OsSAMS1 (S-adenosylmethionine synthase 1), affecting the ethylene synthesis pathway and delaying leaf senescence [123]. Although direct DNA methylation data are lacking, the reduction in SAM content suggests that DNA methylation may regulate leaf senescence. Furthermore, the rice *waf1* (*wavy leaf 1*) (*HEN1* orthologous in *Arabidopsis*) mutant exhibits SAM deficiency, resulting in seedlings mortality and developmental abnormalities, such as wavy leaves and increased leaf angle [124].

Tillering, a unique form of vegetative reproduction in rice, originating from axillary buds at the stem base, and the subsequent panicle formation rate are critical determinants of effective panicle number per unit area, thereby influencing yield composition. The disruption of *OsNRPD1a/b*, the largest subunit genes of Pol IV, leads to a significant reduction in 24-nt siRNA levels. This alteration affects DNA methylation status of MITEs (Miniature inverted-repeat transposable elements), subsequently impacting the expression of nearby genes involved in tiller development, and resulting in increased rice tiller number (Figure 3) [125]. Furthermore, the *rpl1* mutation also exhibits a phenotype of significantly increased tiller number [121].

### 4.2. Reproductive Growth Stage

#### 4.2.1. Heading and Flowering

**Figure 4:**
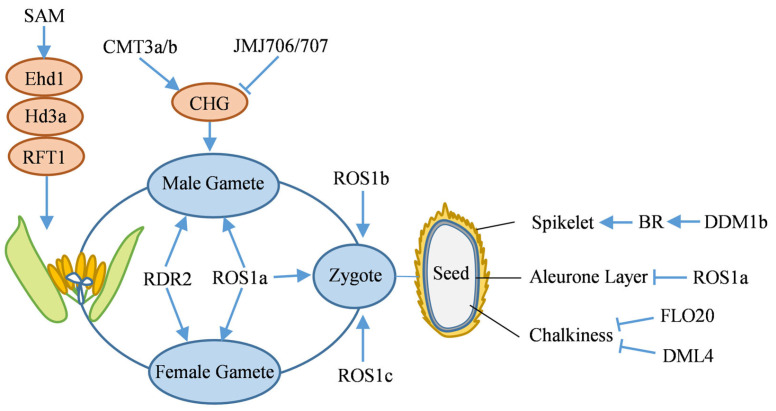
Mechanism of DNA methylation in the reproductive growth stage of rice. Blue arrows and T-bars indicate promotion and inhibition, respectively. Pink ovals represent the increase in DNA methylation levels. BR: Brassinosteroid, CMT3a/b: Chromomethylase 3a/b, DML4: Demeter-like 4, Ehd1: Early heading date 1, FLO20: Floral organ number 20, Hd3a: Heading date 3a, JMJ706/707: Jumonji 706/707, RDR2: RNA dependent RNA polymerase 2, RFT1: Rice flowering locus 1, ROS1a/b/c: Repressor of silencing 1 a/b/c, SAM: S-adenosylmethionine.

The heading stage, a critical transition from vegetative to reproductive growth in rice, is a key factor in determining plant development and yield formation. DNA methylation is also involved in regulating the heading process. Knockdown the rice *SAMS* gene, which encodes S-adenosylmethionine synthetase, leads to a decrease in SAM content, resulting in reduced methylation levels at non-CG sites and histone H3K4me3. These epigenetic changes further suppress the expression of key flowering genes, such as *Ehd1* (*Early heading date 1*), *Hd3a* (*Heading date 3a*), and *RFT1* (*Rice Flowering Locus 1*), ultimately causing delayed heading in rice (Figure 4) [126].

#### 4.2.2. Gamete and Zygote Development

The formation of male and female gametes, along with zygotes development, are central to plant sexual reproduction, directly determining the transmission of genetic material and the development of new individuals. During male gamete development, pollen mother cells undergo meiosis to form microspores, which then generate mature pollen grains containing vegetative cells and sperm cells through mitosis. Female gamete development begins with meiosis of the megaspore mother cell, where the surviving megaspore undergoes three mitotic divisions to form an eight-nucleate embryo sac containing an egg cell. During fertilization, sperm cells enter the embryo sac via the pollen tube: one fuses with the egg cell to form a zygote, while the other fuses with polar nuclei to form an endosperm nucleus [127,128].

A loss-of-function mutation in the *OsROS1a* DNA demethylase gene leads to elevated CG and CHG methylation within the promoter regions of key gametophyte development genes, consequently repressing their expression. The *osros1a* mutant exhibits smaller pollen grains, starch accumulation defects, and iodine-stained sterility, alongside aberrant proliferation of antipodal cells, morphological abnormalities in egg and synergid cells, and partial ovary degeneration post-pollination, resulting in seed abortion (Figure 4) [129]. Additionally, OsDRM2 maintains elevated CHH methylation via the RdDM pathway to ensure proper sexual reproduction [67]. The *osrdr2* mutant impairs 24-nt siRNAs production, leading to diminished CHH methylation, TE activation, and interference with reproductive development gene expression, ultimately causing male and female gamete development abnormalities and sterility (Figure 4) [130].

In rice male gametogenesis, the regulation of DNA methylation is crucial (Figure 4). During the microspore stage, CMT3a/b synergistically enhances CHG methylation, silences TE, and suppresses excessive genome transcription to maintain male gamete genomic stability [59]. In the vegetative cell stage, ROS1a targets promoter and TE regions for local demethylation, and DNA demethylation in vegetative cells can indirectly promote non-CG methylation in sperm [64]. In the sperm stage, histone demethylases JMJ706/707 mediate reduced CHG methylation, activate sperm functional genes, and complete the epigenetic regulatory conversion [59]. 

In rice, DNA methylation orchestrates zygotic gene expression via dynamic demethylation remodeling, thereby establishing a critical epigenetic foundation for typical zygote development and reproductive functions (Figure 4). Research indicates that regional methylation remodeling is initiated within the zygotic genome 6.5 h post-fertilization in rice. Genetic and multi-omics investigations demonstrate that DNA demethylases DNG702 (ROS1a), DNG701 (ROS1b), and DNG704 (ROS1c) facilitate DNA demethylation across diverse genomic regions in gametes and zygotic cells. These demethylases are essential for activating zygotic gene expression and maintaining developmental processes [63].

#### 4.2.3. Seed Development

The primary attributes of rice seeds encompass grain shape (affecting yield) and quality (determining edibility). Grain shape is characterized by metrics such as grain length, width, and thickness, while quality is assessed through appearance, milling properties, taste, and nutritional content [131].

Previous studies have demonstrated that dynamic DNA methylation regulation is critical in the developmental process governing rice grain shape (Figure 4). The rice chromatin remodeling factor OsDDM1b (Decrease in DNA methylation 1b) maintains heterochromatin DNA methylation via ATP-dependent nucleosome remodeling, which subsequently regulates cell cycle genes to promote glume cell division. Additionally, it may modulate brassinosteroid (BR) homeostasis and signal transduction by maintaining methylation. The loss function of *OsDDM1b* results in cell cycle arrest, reduced BR content, and significantly smaller grains [61]. Furthermore, DNA methylation plays a crucial role in regulating rice seed quality by influencing the epigenetic modification status of genes involved in storage proteins and starch synthesis, as well as affecting nutrient accumulation and distribution during endosperm development (Figure 4). The FLO20 (Floral organ number 20), encoding SHMT4 protein, interacts with SAMS2 to modulate SAM synthesis. This interaction maintains the SAM/SAH (S-adenosyl-l-homocysteine) balance, which is crucial for DNA methylation, and subsequently regulate the expression of starch and storage protein genes. In the *flo20* mutant, a reduction in SAM concentration induces elevated genome-wide DNA methylation, thereby repressing the expression of key transcription factors (TFs) involved in starch and storage protein synthesis. This ultimately results in endosperm chalkiness, disrupted starch granules, and aberrant protein bodies (PBs) [132]. *OsDML4* regulates the expression of storage protein genes via DNA methylation. Functional deficiency of *OsDML4* results in elevated CG, CHG, and CHH methylation levels in the endosperm under high temperature conditions, leading to hypermethylation and downregulation of storage protein-related genes. This, in turn, causes aberrant PBs formation and disrupted starch granule arrangement, ultimately increasing grain chalkiness [66].

A dominant negative mutation in the DNA demethylase gene *OsROS1* leads to an expansion of the aleurone layer in the wild type to multiple layers in the mutant (Figure 4). This specific mutation causes aberrant splicing, leading to increased CG and CHG methylation in the endosperm. Two aleurone layer differentiation-related TFs are hypermethylated and exhibit reduced expression, promoting an increase in aleurone layer cell numbers and enhancing the content of non-starch nutritional components [62].

## 5. Role of DNA Methylation in Rice Stress Response

### 5.1. Abiotic Stress

Rice is frequently exposed to abiotic stresses during its growth and development, which can significantly affect its physiological metabolism, growth status, and yield quality. Common abiotic stresses include high/low temperature, drought, salinity, heavy metal, and nutrient stresses [14].

**Figure 5:**
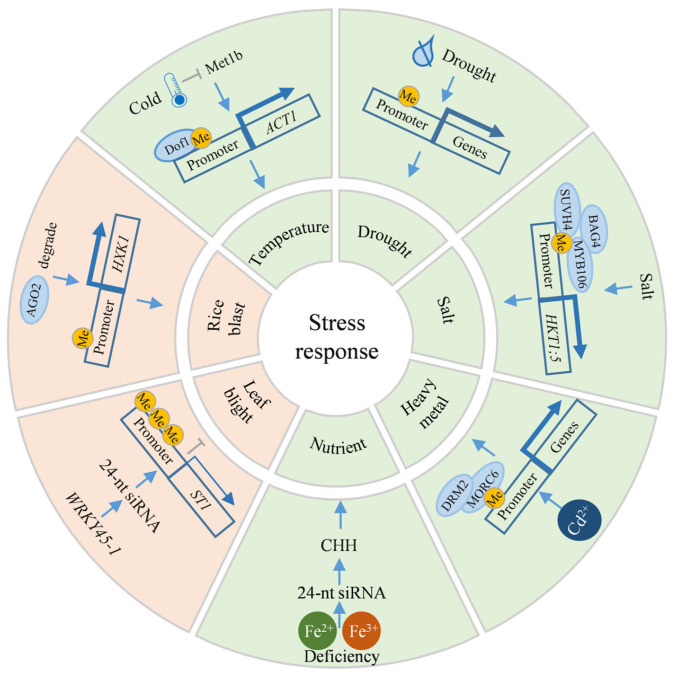
Mechanism of DNA methylation in stress resistance processes. Blue arrows and gray T-bars indicate promotion and inhibition, respectively. Pink and green regions correspond to biotic and abiotic stresses, respectively. A single yellow “Me” marker represents a low methylation level, while three yellow “Me” markers represent a high methylation level. Thick blue arrows indicate high expression, and thin blue arrows indicate low expression. AGO2: Argonaute 2, BAG4: Bcl-2-associated athanogene protein 4, Dof1: DNA binding with one finger 1, DRM2: Domains rearranged methyltransferase 2, HKT1;5: High-affinity potassium transporter 1;5, HXK1: Hexokinase 1, Me: Methylation, Met1b: Methyltransferase 1b, MORC6: Minichromosome maintenance 1-related protein 6, ST1: Susceptibility to blast 1.

Temperature is a critical environmental determinant affecting rice production. Analyzing the response mechanisms of rice to both high and low temperature stresses can provide genetic resources for breeding temperature-tolerant rice varieties. Guo et al. observed that in the cold-tolerant rice variety P427, the number of genes exhibiting DNA methylation alterations under low-temperature stress was significantly higher compared to other varieties. Specifically, the expression of certain genes is significantly upregulated due to reduced DNA methylation, potentially playing a key role in the cold tolerance of P427 [133]. Additionally, the cold tolerance conferred by DNA methylation in rice demonstrates stable genetic characteristics. Song et al. indicated that low-temperature stress can suppress the expression of the DNA methyltransferase gene *MET1b*, resulting in DNA demethylation within the promoter region of the *ACT1* (*Acquired cold tolerance 1*) gene (Figure 5). This demethylation facilitates the binding of the upstream Dof1 (DNA binding with one finger 1) transcription factor, which subsequently activates *ACT1* expression and enhances rice cold tolerance. The hypomethylated region in the *ACT1* promoter represents a key domestication site for rice cold adaptation, and cold-tolerant lines carrying this epigenetic modification can be stably inherited for at least five generations under normal temperature conditions (Figure 5) [20]. Conversely, high temperature stress during rice grain development negatively impacts the synthesis of various substances, leading to increased chalkiness. Research has confirmed that under high-temperature stress conditions, the genome-wide DNA methylation is significantly increased in the *osdml4* mutant, and the content of gluten and alcohol-soluble proteins decreases, ultimately resulting in a chalky endosperm phenotype [65].

Drought stress poses a major abiotic constraint on rice production, with roughly half of the world’s rice-growing regions experiencing seasonal drought, resulting in an average yield reduction of 30–50% [134]. Drought treatment experiments on drought-tolerant and drought-sensitive rice varieties have shown that drought-tolerant varieties display a hypomethylated status under drought stress, which correlates with the high expression of some abiotic stress-responsive genes [110]. Differentially methylated regions (DMRs) linked to rice drought stress memory exhibit dynamic and unique patterns of change [135]. Similarly, epigenetic mutations induced by drought treatment in rice can perpetuate the altered DNA methylation status in subsequent generations, with genes associated with transgenerational epigenetic mutations directly involved in drought stress responses (Figure 5) [136].

Soil salinity poses a substantial constraint on global agricultural practices, especially in rice cultivation, where salinization can impede rice development and significantly diminish yield. Unlike drought stress, salt stress conditions induce elevated DNA methylation in salt-tolerant varieties compared to sensitive ones. This observation correlates with the upregulated expression of specific genes responsive to abiotic stressors [110]. Wang et al. elucidated a mechanism wherein a complex comprising a DNA methylation recognition enzyme, a chaperone regulatory protein, and a TF modulates the expression of *OsHKT1;5* (*High-affinity potassium transporter 1;5*) to mediate salt stress response: the DNA methylation recognition enzyme OsSUVH7 recognizes CHG and CHH methylation of the MITE element upstream of *OsHKT1;5*. Simultaneously, OsBAG4 (Bcl-2-associated athanogene protein 4) facilitates the interaction between OsSUVH7 and OsMYB106, thereby prompting the binding of OsMYB106 to the upstream region of *OsHKT1;5* and activating *OsHKT1;5* expression in response to salt stress (Figure 5) [137]. OsDML4 has been identified as a participant in the rice salt stress response. The *osdml4* mutant exhibits increased sensitivity to salt stress, characterized by aberrant ROS accumulation and an elevated Na⁺/K⁺ ratio, potentially influencing ROS homeostasis and the jasmonic acid (JA) signaling pathway [65].

The cultivation of rice in heavy metal-contaminated soil presents multifaceted challenges, impacting not only plant growth, yield, and quality, but also posing risks to human health via the food chain. Tan et al. indicates that the rice Microrchidia family protein OsMORC6 (Minichromosome maintenance 1-related protein 6) recruits OsDRM2 through the RdDM pathway to facilitate DNA methylation, thereby positively influencing rice’s chromium tolerance (Figure 5) [138]. Furthermore, heavy metal stress-induced alterations in DNA methylation patterns can be stably inherited in rice, enhancing heavy metal tolerance in subsequent generations [139]. 

Nutrient stress, encompassing both macro- and micronutrient imbalances, significantly affects rice’s physiological metabolism, yield formation, and quality. Sun et al. observed that under iron-deficient conditions, the abundance of 24-nt siRNA increases, accompanied by elevated genome-wide CHH methylation, particularly in TE regions in rice (Figure 5). This methylation change suppresses TE activity and activates adjacent iron-deficiency response genes, thereby improving rice’s adaptability to iron-deficient conditions [140].

The aforementioned environmental stressors, including cold, drought, and heavy metals, have been demonstrated to trigger transgenerational epigenetic memory in rice. In fact, plants frequently exhibit transgenerational epigenetic memory under abiotic stress [141]. Current research indicates that the preservation and inheritance of epigenetic modifications underpin this phenomenon at the molecular level. During germ cell formation and zygotic development, the genome undergoes DNA methylation reprogramming, whereas DNA methylation status of specific loci can resist this reprogramming and remain stable, acting as transgenerational memory carriers [118]. Histone modifications can prevent DNA methylation resetting via the RdDM pathway. For example, H3K4me3 directly inhibits the recruitment of core components in the RdDM pathway, while H3K18ac actively removes DNA methylation by recruiting DNA demethylases. This dual mechanism of inhibiting RdDM activity preserves the transgenerational stability of the initial epigenetic states [118]. Small RNAs can also influence DNA methylation inheritance. In maize, 24-nt siRNAs can be transmitted transgenerationally and induce CHH methylation via the RdDM pathway, which can then be converted into a stable and inheritable state [142]. 

### 5.2. Biotic Stress

Current investigations into biotic stress in rice primarily emphasize the fungal disease rice blast and the bacterial disease bacterial leaf blight. Rice blast, incited by *Magnaporthe oryzae*, can infect rice throughout its entire development stages, from the spindle-shaped lesions of leaf blast during the seedling phase to white panicles caused by panicle neck blast, frequently leading to a 40-50% yield reduction [143]. Early studies indicated that the expression of the rice blast resistance gene *Pib* is significantly upregulated upon exposure to the rice blast fungus, with its core promoter region exhibiting a highly CG-methylated state. Notably, promoter region demethylation leads to reduced rice blast resistance [144]. Another study revealed that OsAGO2, a critical component of the silencing complex in the rice RdDM pathway, participates in the rice blast resistance response. In the absence of rice blast fungus infection, OsAGO2 binds to miR1875 and suppresses the expression of the target gene *OsHXK1* (*Hexokinase 1*) by methylating its promoter region via DNA methylation (Figure 5). Upon infection by the rice blast fungus, OsAGO2 is degraded, releasing the inhibition of *OsHXK1* expression, thereby enhancing the plant’s resistance to the disease [145]. Additionally, a novel miR812w originating from TEs in rice, is crucial for immunity against rice blast disease. miR812w modulates host defense genes via both *cis* and *trans* DNA methylation. These regulations induce CHH hypermethylation of target genes, thereby inhibiting their expression, which subsequently suppresses pathogen infection and positively regulates rice resistance to the rice blast fungus [146]. Deng et al. discovered that the *Pigm* locus harbors two antagonistic NBS-LRR-type receptor proteins, PigmR and PigmS. These proteins regulate the CHH methylation of their promoter regions via RdDM pathway, ensuring that *PigmR* is expressed primarily in tissues like leaf, while *PigmS* is highly expressed in pollen. This epigenetic regulatory mechanism dynamically balances the relationship between disease resistance and yield. Upon pathogens invasion, PigmR governs the disease resistance response in leaves, during reproductive development, PigmS in pollen maintains yield by enhancing fertility [147].

Bacterial leaf blight, incited by *Xanthomonas oryzae*, manifests as characteristic yellow-white lesions that progress along leaf margins. Zhang et al. identified two *WRKY45* alleles, *WRKY45-1* and *WRKY45-2*. A TE insertion within the *WRKY45-1* intron leads to the generation of multiple, partially overlapping 24-nt siRNAs from its transcript. These siRNAs trigger CHH methylation in the promoter region of *ST1* (*Susceptibility to blast 1*) gene, a key element in the downstream disease resistance signaling pathway, via the RdDM pathway, consequently suppressing *ST1* expression and ultimately compromising rice’s resistance to blast disease (Figure 5). In contrast, *WRKY45-2*, lacking TE insertion, permits normal *ST1* expression, thereby conferring resistance to bacterial leaf blight in rice [148].

## 6. Role of DNA Methylation in Other Biological Process

In rice, DNA methylation is implicated in the regulation of growth and development processes, and stress responses, and also plays a crucial role in significant biological processes, including domestication and heterosis formation, through dynamic epigenetic modifications.

Rice domestication is characterized by the anthropogenic conversion of wild rice into cultivated rice through sustained human selection. Conversely, de-domestication denotes the phenomenon where specific rice varieties gradually revert to some phenotypic traits of wild rice, evolving into weedy rice [149]. A comprehensive methylome analysis of wild, cultivated, and weedy rice revealed a significant decrease in genome-wide DNA methylation during rice domestication, contrasting with a marked increase during de-domestication. The methylation variation regions associated with domestication and de-domestication do not overlap, suggesting that de-domestication is not a simple reversal of domestication. Furthermore, distinct DNA methylation patterns have been observed among various cultivated rice subtypes [112]. A separate investigation into the genome-wide DNA methylation, transcriptome, and metabolome of japonica and indica rice germplasms revealed that the genome-wide DNA methylation of indica rice is notably lower than that of japonica rice [150]. Peng et al. observed that under natural conditions, an active retrotransposon HUO is prevalent in wild rice, whereas it is absent in cultivated rice varieties. HUO influences functional genes at the genomic level via the RdDM pathway, thereby activating the genome’s defense mechanisms. This activation facilitates wild rice’s adaption to intricate and dynamic natural environments; however, it is not conducive to high and stable yields in cultivated rice. Therefore, it has been gradually and selectively eliminated during the domestication and breeding of rice [151].

The manifestation of heterosis in rice is intricately linked to DNA methylation. Zhou et al. revealed that the overall DNA methylation in hybrid rice seeds is reduced compared to their parents, a factor critical for the onset and persistence of hybrid vigor [152]. Ma et al. demonstrated an inverse relationship between CHG methylation and allelic-specific expression (ASE), with the CMT3 enzyme implicated in this regulatory mechanism. In addition, non-additive CHG methylation regions are abundant in genes associated with hormone signaling pathways, potentially contributing to the enhanced environmental adaptability observed in hybrid rice cultivars [153].

## 7. Summary and Prospects

DNA methylation is widely recognized as a pivotal regulatory mechanism influencing rice growth and development, stress response, domestication, and heterosis. Nevertheless, current studies present several limitations. Firstly, despite the known involvement of various DNA methylation-related enzymes in regulation, the precise molecular mechanisms by which these enzymes specifically regulate methylation sites and levels across diverse biological processes remain incompletely elucidated. For instance, in response to intricate and fluctuating natural environmental conditions, the adaptive strategies of rice, mediated by dynamic alterations in DNA methylation status through the regulation of related enzyme activity, require further investigation into the signaling pathways and key regulatory nodes involved. Secondly, although certain DMRs and genes associated with significant traits have been identified, the causal relationships between these DMRs and gene expression, as well as their synergistic regulation of rice phenotypes, necessitate extensive functional verification experiments. Finally, the interplay among different epigenetic regulatory mechanisms, including DNA methylation, histone modification, and non-coding RNA regulation, along with their integrated regulatory networks in complex rice biological processes, remains poorly understood.

Future advancements in technology will facilitate more precise and comprehensive analysis of the rice DNA methylation landscape and its dynamic alterations. This will involve investigating key regulatory genes and molecular modules governing DNA methylation in rice growth, development and stress response. Furthermore, employing gene editing and related technologies to precisely modulate rice DNA methylation status will enable the breeding of novel rice varieties with enhanced yields, quality and stress tolerance. To expedite the attainment of the aforementioned objectives, several technical hurdles in DNA methylation research necessitate immediate resolution. Current methodologies for DNA methylation detection continue to grapple with the inherent "triple trade-off" encompassing coverage, resolution, and cost, with no single technology universally applicable across all experimental contexts [154,155]. Additionally, functional validation of differentially methylated sites heavily relies on targeted editing technologies, which present notable limitations. Specifically, the editing efficiencies in plants are relatively low, ranging from 10% to 30% [156,157,158,159,160]. Both issues impede large-scale functional verification of candidate sites. Moreover, these technologies are currently unable to disentangle the specific contributions of DNA methylation alterations from other epigenetic modifications, such as histone modifications, thereby complicating the attribution of functional outcomes [161,162].

With the ongoing progress in DNA methylation research, breeders are increasingly exploring the application of DNA methylation-based regulatory strategies in epigenetic breeding. Utilizing epigenetic editing technologies, researchers can precisely modulate methylation levels in the promoter regions of specific DNA methylation sites, thereby enabling the development of stress-tolerant rice varieties. A targeted DNA demethylation system based on SunTag-dCas9-TETcd was developed in rice, enabling precise regulation of methylation within the promoter region of the *OsFIE1* gene with stable inheritance across generations [163]. Furthermore, targeted editing of the methylation status of the *ACT1* gene has enabled the directed modulation of cold tolerance in rice [20]. By leveraging epigenetic marker-assisted selection (EMAS) technology, researchers can screen for materials or loci associated with target traits. Methylation quantitative trait loci (meQTL), which serve as the foundation for screening valid markers in EMAS, are currently a significant research focus. meQTLs, defined as genetic variants exhibiting statistically significant associations with specific DNA methylation patterns, can be identified through large-scale population DNA methylation analyses combined with linkage mapping of genetic variants [94]. Similar meQTL analyses have been performed in plants such as tomato, pear, and cotton [94,164,165]. Despite the existence of population-level DNA methylation studies in rice, linkage analyses correlating DNA methylation with genetic variants remain unexplored, which could provide guidance for future applications of DNA methylation in rice breeding [150,151,152,153].

Theoretically, a comprehensive understanding of DNA methylation mechanisms in rice clarifies how such epigenetic modifications dynamically regulate genes associated with yield, quality, and stress tolerance. Practically, unraveling these mechanisms establishes a foundational basis for innovative breeding technologies. In conclusion, in-depth investigation of the mechanism of DNA methylation in rice is of significant theoretical and practical importance for advancing rice genetic improvement and ensuring global food security.

## Figures and Tables

**Table 1:** Some cloned DNA methylation-related genes in rice.

No.	Gene in Rice	Function Analysis	Reference
1	*OsDMR2*	Plant height, fertility, number of tillers	[57]
2	*OsCMT3a*	Plant height, heading time, grain filling rate, zygote development	[58,59]
3	*OsCMT3b*	Zygote development	[59]
4	*OsMET1a*	/	[60]
5	*OsMET1b*	/	[60]
6	*OsDDM1a*	Plant height, grain filling rate	[57]
7	*OsDDM1b*	Internode length, fertility, spike length, fruit set rate, grain shape, organ size	[61]
8	*OsROS1a*	Endosperm components, germ cell development, root development, zygote development	[62,63,64]
9	*OsROS1b*	Zygote development	[63]
10	*OsROS1c*	Zygote development, embryo development	[63]
11	*OsDML4*	Heat resistance, endosperm components	[65,66]
